# “Acute urinary antibiotics”—A simple metric to identify outpatient antibiotic stewardship opportunities in renal transplant

**DOI:** 10.1017/ash.2023.330

**Published:** 2023-09-29

**Authors:** Alex Zimmet, David Ha, Emily Mui, Mary Smith, William Alegria, Marisa Holubar

## Abstract

**Background:** *International Classification of Diseases, Tenth Edition* (ICD-10) data help track outpatient antibiotic prescribing but lack validation in immunocompromised populations or subspecialty clinics for this purpose. Asymptomatic bacteriuria (ASB) and urinary tract infection (UTI) are important stewardship targets in renal transplant (RT) patients, but they may require alternative metrics to best monitor prescribing patterns. We describe ICD-10 utilization for RT clinic encounters in which antibiotics were prescribed. We developed a metric classifying “acute urinary antibiotics” (AUA) to track antibiotic use for ASB and UTI, and we validated systematic identification of AUA to enable practical implementation. **Methods:** We examined RT clinic visit and telemedicine encounters from 2018 to 2021 conducted 1 month after transplant. This project was deemed non–human-subjects research by the Stanford Panel on Human Subjects in Medical Research. **Results:** The analytic cohort included 420 antibacterial prescriptions from 408 encounters (Fig. 1). Of 238 patients, 136 (57%) were male and 112 (47%) were Hispanic or Latino. The most common primary ICD-10 code was Z94.0 (kidney transplant status) (N = 302 of 408 encounters, 75%); 26 encounters (6%) were coded for UTI (eg, N39.0, urinary tract infection, site not specified); and 214 encounters (53%) had multiple ICD-10 codes. The R82.71 code (bacteriuria) was never used. However, 215 prescriptions (51%) were classified as AUA (Fig. 2). The validation cohort included 130 prescriptions; 59 (45%) were classified as AUA and 51 (39%) had documented intent to treat ASB or UTI (positive percent agreement, 83%; negative percent agreement, 97%) (Table 1). For patients >1 month after transplant, the positive percent agreement was 95% and the negative percent agreement was 98%. Of 51 patients receiving AUA, 32 (63%) were asymptomatic despite frequently having a code for UTI (Fig. 3). **Conclusions:** ICD-10 coding may not be helpful in monitoring antibiotic prescribing in RT patients. The AUA metric offers a practical alternative to track antibiotic prescribing for urinary syndromes and reliably correlates with physician intent. Monitoring AUA prescribing rates could help identify opportunities to optimize antibiotic use in this complex outpatient setting.

**Figure:**
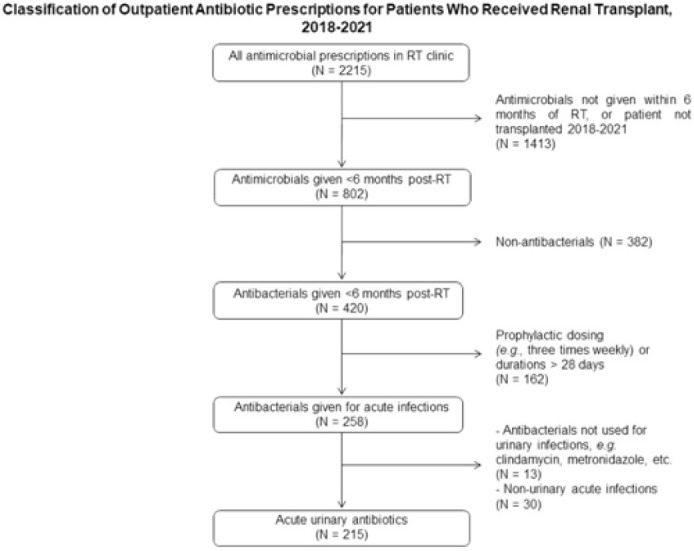

**Figure:**
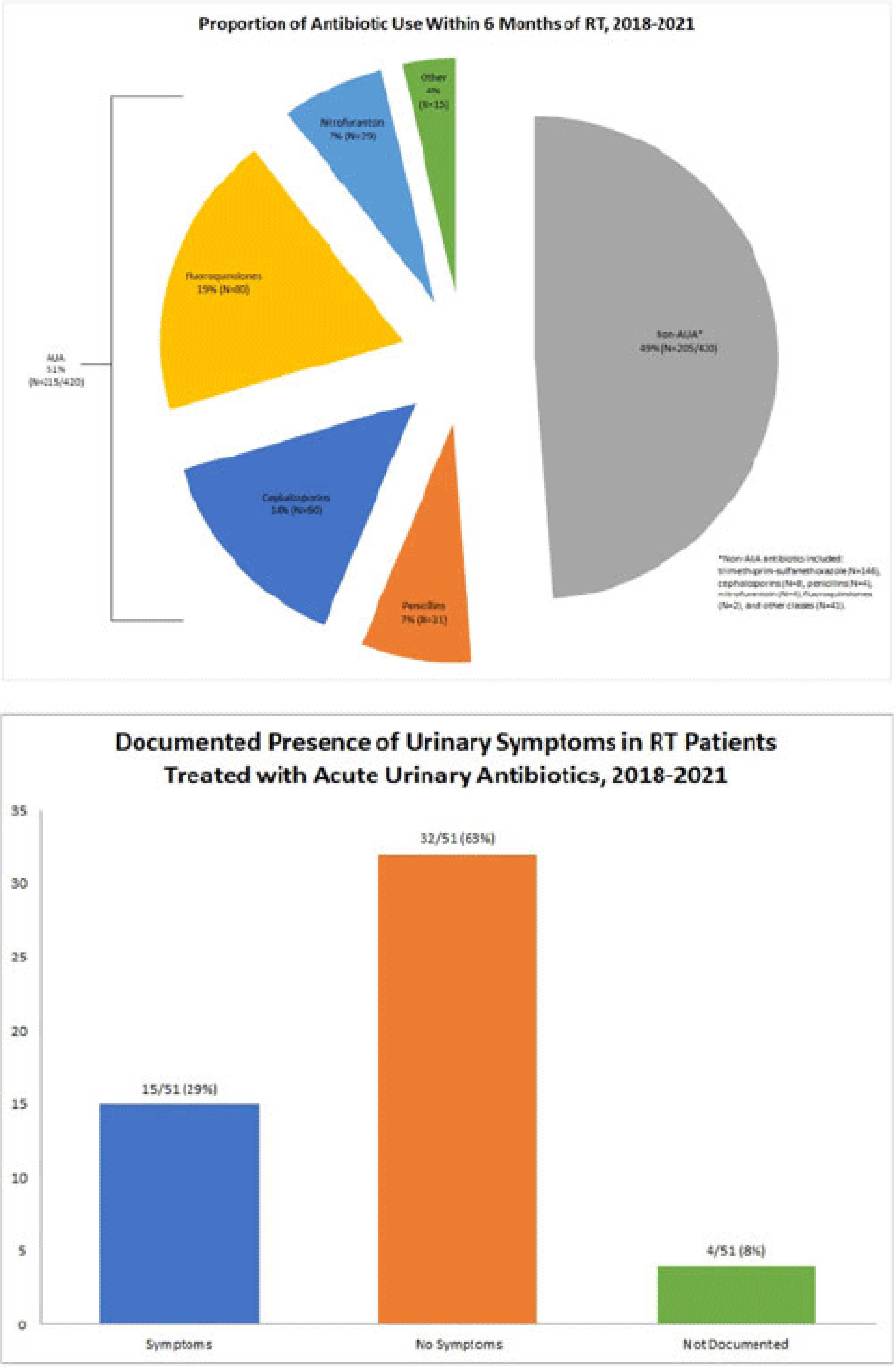

**Figure:**
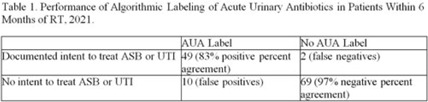

**Disclosures:** None

